# Antitumor functions and mechanisms of nitidine chloride in human cancers

**DOI:** 10.7150/jca.37890

**Published:** 2020-01-01

**Authors:** Yue Cui, Linhui Wu, Ruoxue Cao, Hui Xu, Jun Xia, Z Peter Wang, Jia Ma

**Affiliations:** 1 Research Center of Clinical Laboratory Science, School of Laboratory Medicine, Bengbu Medical College, Anhui, China, 233030, China.; 2 Department of Laboratory Medicine, School of Laboratory Medicine, Bengbu Medical College, Anhui, 233030, China.; 3 Department of Biochemistry and Molecular Biology, School of Laboratory Medicine, Bengbu Medical College, Anhui, 233030, China.; 4 Department of Pathology, Beth Israel Deaconess Medical Center, Harvard Medical School, Boston, MA, USA.

**Keywords:** Nitidine chloride, Cancer, Therapy, Target, Antitumor

## Abstract

Nitidine chloride (NC), a quaternary ammonium alkaloid, exhibits multiple biological activities, including antimalarial, antifungal, and antiangiogenesis. Recently, NC has been characterized to perform antitumor activity in a variety of malignancies. NC has been identified to suppress cell proliferation, stimulate apoptosis, and induce cell cycle arrest, retard migration, invasion and metastasis. Moreover, NC is reported to sensitize cancer cells to chemotherapeutic drugs. In this review article, we describe the functions of NC in human cancers and discuss the molecular insight into NC-involved antitumor feature. This review article will stimulate the deeper investigation for using NC as a potent agent for the management of cancer patients.

## Introduction

**Figure 1:**
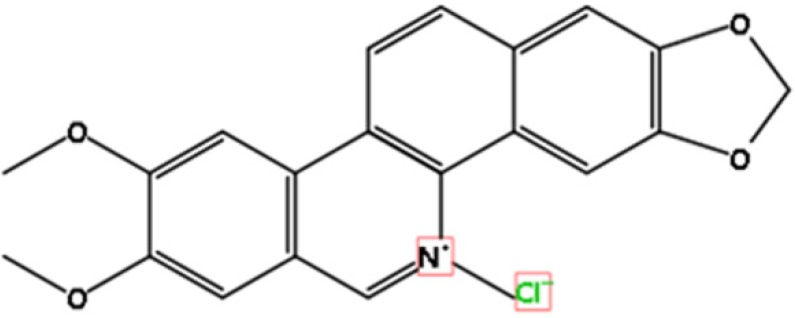
The structure of Nitidine chloride is illustrated. The molecular formula is C21H18CLNO4.

**Figure 2:**
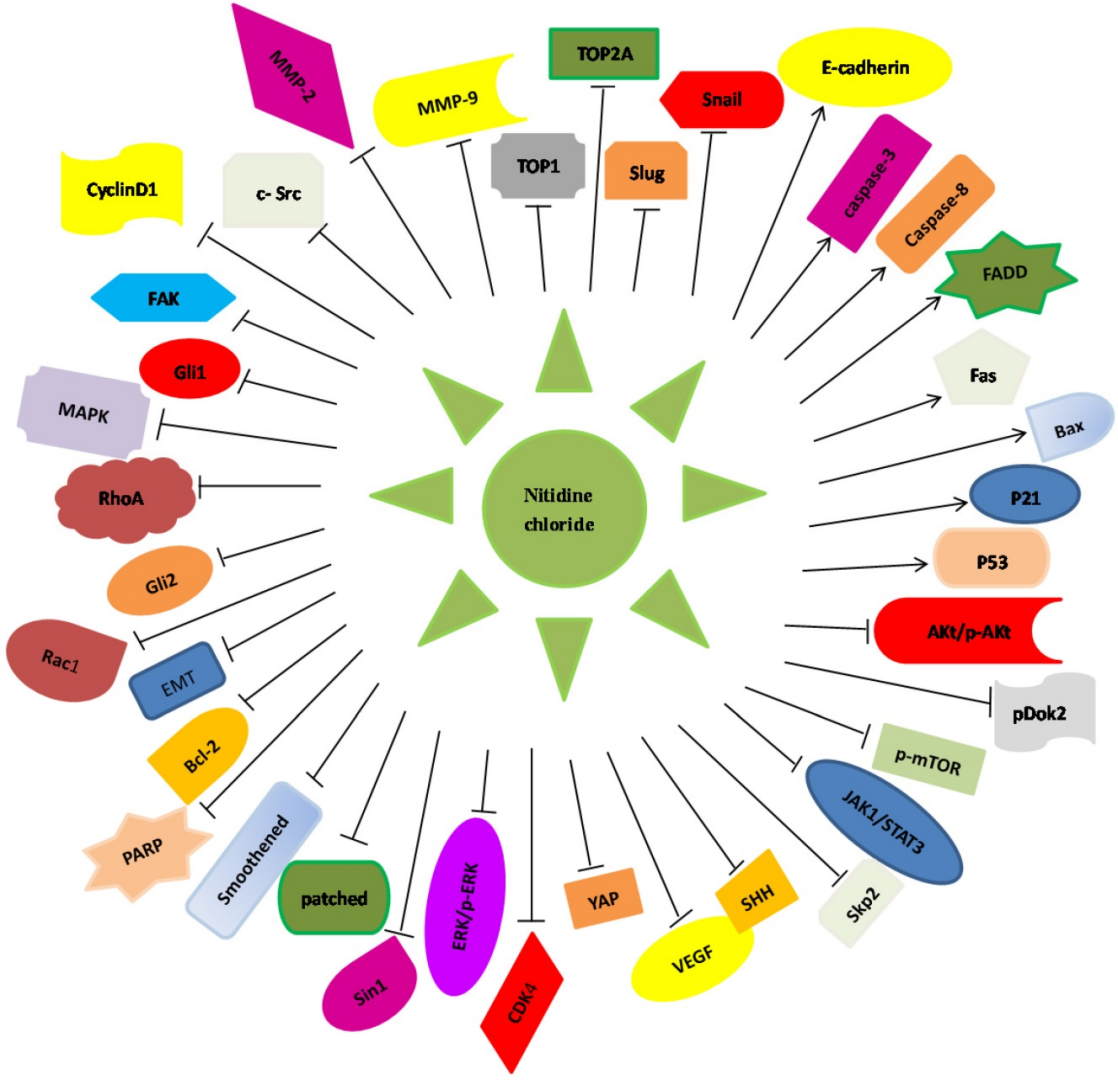
Illustration of Nitidine chloride -regulated targets and signaling pathways in human cancers. Nitidine chloride exerts its anti-tumor function via regulating the expression of these downstream genes in human malignancies.

Nitidine, a phytochemical alkaloid, is mainly extracted from the root of Zanthoxylum nitidum that belongs to the family of Rutaceae, which is often distributed in Northeastern Asia 1 (Figure 1). Nitidine has been characterized as a folk medicine because it is helpful for reducing pain, enhancing blood circulation and cleaning blood stasis 1. Some studies have revealed that nitidine has effects on protection of tumor, inflammation, and HIV infection 2, 3. NC exhibits multiple biological activities including antimalarial 4, antifungal 5, and antiangiogenesis 6. Moreover, NC has been identified to inhibit cell proliferation, induce apoptosis, trigger cell cycle arrest, and sensitize cancer cells to chemotherapeutic drugs. Some researchers have discovered the underlying antitumor mechanisms of NC in human cancers 1, 7. In this review article, we describe the functions of NC in human cancers and highlight the molecular insight of NC-induced antitumor feature (Figure 2).

## Role of NC in human cancers

### Breast cancer

Breast cancer is one of the leading causes of cancer-mediated deaths in female 8. Currently, the treatments of breast cancer include surgery, radiotherapy, and chemotherapy. However, due to metastasis and drug resistance, breast cancer patients have poor prognosis 8. NC has been reported to inhibit the metastasis of breast cancer cells via inactivation of the c-Src/FAK-associated pathway 3. Moreover, NC decreased the MMP-9 and MMP-2 formation and their proteolytic activity in mammary cancer cells 3. Mechanistically, NC reduced PDGF-triggered phosphorylation of c-Src, FAK, MAPK, and inactivated the activity of RhoA, Rac1, and AP 3. Another study revealed that NC exposure led to inhibition of cell proliferation and induction of cell cycle arrest through elevation of multiple gene expressions such as p53, p21, Bax, and active forms of caspase-3, caspase-9, and cleaved PARP, and downregulation of Bcl-2 9. Notably, NC sensitizes cell sensitivity to doxorubicin for cell proliferation in breast cancer 9.

Accumulating evidence suggest that EMT plays an essential role in tumor metastasis due to that mesenchymal cells acquire more migratory feature 8. EMT is a programme that epithelial cells transfer to mesenchymal cells, which is often happened in multiple biological processes such as fibrosis, wound healing, and tumor metastasis 10. The cell phenotype is changed from apical-basal polarity and tight junctions to elongated and spindle-shape cells and loose interaction, leading to increased migration and invasion 11. EMT molecular markers are changed from loss of epithelial markers such as E-cadherin to acquired mesenchymal molecules such as Slug, Snail, Vimentin, Zeb1, and Zeb2 12. Various EMT inducers led to EMT in cells, which have CSC characters 13, 14. CSC have been identified in multiple types of cancers, which is associated with tumor metastasis, drug resistance, and tumor reoccurrence 15. CD44^+^/CD24^-^ has been characterized as a marker for breast CSC, and CSC are involved in tumor metastasis and radiotherapy resistance 16, 17. Recently, Sun et al. identified that NC inhibited migratory and invasive capability due to suppression of EMT and CSC-like phenotype by suppression of HH pathway in breast cancer cells 18. Specifically, NC downregulated the expression of several molecules in HH pathway including Gli1 and Gli2, suppressed the expression level of mesenchymal markers such as Zeb1, Slug, and Snail, leading to EMT reversal. Strikingly, NC attenuated the expression of Nanog, Nestin, Oct-4 and CD44 through HH pathway in breast cancer cells 18. These reports indicate that NC exerts its tumor suppressive function in breast cancer cells.

### Liver cancer

LC is one of the common malignancies, with HCC as a main subtype 19. The pathogenesis of HCC and its development process are complex and are related to a variety of signal transduction pathways, including STAT3, SHH, and ERK 20. One study reported that the NC repressed HCC proliferation though JAK1/STAT3 pathway due to enhancement of apoptosis and increased level of p21 and Bax, and decreased cyclinD1, CDK4 and Bcl-2 in HCC 21. Similarly, another study also found that NC reduced tumor volume and tumor weight in mice via suppression of ERK, STAT3, and SHH pathways, and regulation of Bcl-2, Bax, CyclinD1, VEGF pathway 20. These genes are involved in cell proliferation, apoptosis, and tumor angiogenesis in liver cancer 20.

In addition, Ou et al also found that NC affected cell proliferation, apoptotic death, and cell cycle by elevation of p53, Bax, caspase-3 and p21, but inhibition of Bcl-2 in liver cancer 1. Furthermore, a supramolecular formulation of NC has been developed and alleviated hepatotoxicity and improved anticancer activity 22. Interestingly, NC was found to be a candidate substrate of OCT1 and OCT3 that transfer NC into hepatocytes. Moreover, MATE1 makes NC leave from hepatocytes, while CYPs contributed to NC metabolism such as CYP3A4 and attenuated the hepatic toxicity of NC 23. TOP1 and TOP2A are key tumor drivers in liver cancer 24, suggesting that TOP1 and TOP2A might be promising targets for treating malignances 25. One group has shown that the NC exposure attenuated the TOP1 and TOP2A expression levels 26. Recently, NC was found to suppress the expression of eight genes that are highly expressed in liver cancer 27. The analogues of nitidine have been synthesized and exhibited extraordinary inhibition of cell proliferation in liver cancer cells 28. Similarly, supramolecular formulation of NC reduced its toxicity to liver and promoted tumor suppressive activity in liver cancer cells 29. However, further experiments are necessary to define the molecular mechanism of NC in HCC.

### Ovarian cancer

OC is one type of malignancies in females. The Fas/FasL system is involved in carcinogenesis 30. Fas can bind to FasL and initiate activation of caspase-8 and caspase-3 to exert apoptosis 31. Fas expression was remarkably downregulated in OC patient clinical samples 32, 33. Therefore, overexpression of Fas could become a potential therapeutic target for OC 34. NC was found to elevate the level of FADD, caspase-8 and caspase-3 in OC cells 34. Skp2 as an oncoprotein is the key adaptor of the SCF type of E3 ligase 35-37. NC was reported to inhibit the Skp2 level in OC cells, while Skp2 upregulation abolished NC-induced antitumor property 38. Thus, suppression of Skp2 expression level by NC might be a useful approach for management of OC patients 38. There is one study showing that NC blocked cell migratory and invasive activity through downregulation of MMP-2 as well as MMP-9 via suppression of ERK pathway in OC cells 39. NC induced apoptosis and retarded cell proliferation in OC cells by reduced pAkt and modulation of Bcl-2 family expression 40. Moreover, NC sensitized OC cells to doxorubicin treatment, leading to a synergistic suppression of proliferation 40.

### Renal cancer

RCC patients with distant metastasis have low five-year survival rate. The underlying molecular mechanisms of renal cancer development and progression are unclear. The higher expression of pAkt is exhibited in RCC, especially in later and metastatic RCC 41, 42. One study has shown that NC attenuated the cell invasion and metastasis of RCC cells through inhibition of Akt signaling pathway and MMP-2 and MMP-9 43. This group further found that NC inhibited phosphorylation of ERK and Akt, upregulated the protein level of p53, Bax, cleavage caspase-3 and PARP and downregulated Bcl-2 in RCC cells 44. Without a doubt, the detailed molecular insight into NC-induced antitumor activity in RCC is required to be further investigated.

### Glioblastoma

GBM is one type of aggressive brain malignancy 45. Due to that GBM is highly proliferative, invasive and chemoresistant, the combined treatments of surgery, chemotherapy, and radiotherapy have not significantly improved the survival rate of GBM patients 46, 47. Liu et al dissected that NC impaired cell viability and motility of GMB cells via inactivation of pAkt and mTOR 48. Moreover, NC was reported to increase the expression of cleaved PARP and cleaved caspase 3, and to inhibit pDok2 in GBM cells 49. Similarly, NC prohibited cell proliferation and colony formation, induced cell cycle arrest at G2/M phase in glioma cells. Notably, NC suppressed GSK3-β pathway in glioma cells 49. Taken together, NC could be a potential anti-glioma agent, which is needed to further explore its antitumor activity.

### Osteosarcoma

Osteosarcoma is the common bone malignant tumor in young adults and children 50-52. One study reported that NC prevented the growth of osteosarcoma cells and also stimulated the apoptosis via elevated cleaved forms of caspase-3 and caspase-9, and increased Bax, and down-regulation of Bcl-2 53. Another study revealed that NC inhibited EMT process and suppressed the invasive ability via targeting the Akt/GSK-3β/Snail signaling pathway 54. Specifically, NC treatment elevated E-cadherin expression and repressed the level of N-cadherin, vimentin, and fibronectn in osteosarcoma cells 54. Recently, Xu et al demonstrated that NC impeded proliferation, migration, and invasion, and stimulated apoptosis by inhibition of SIN1 in osteosarcoma cells, indicating that NC might be a useful agent to act as an inhibitor of SIN1 in osteosarcoma 55. Altogether, NC could be a potential agent for treating osteosarcoma.

### Other cancers

CRC is one of the most commonly diagnosed malignancies. NC was identified to impede the proliferation of CRC cells and induce apoptosis 56. NC treatment in CRC cells promoted the protein level of Bax, p53, cleaved caspase-3 and -9, and decreased Bcl-2 level and reduced the phosphorylation of ERK, which could lead to cell proliferation inhibition and apoptosis 56. Elevation of STAT3 expression is observed and associated with metastasis in GC 57. NC inhibited STAT3 activation, leading to repression of its downstream targets such as cyclin D1, Bcl-xL, and VEGF in GC cells 6. This finding dissects that NC could be a potent STAT3 inhibitor in gastric cancer to treat this deadly disease. However, further research is necessary to define the mechanism of NC in suppression of growth and metastasis of GC cells.

NC inhibited cell viability via induction of apoptosis by hindering STAT3 pathway in oral cancer cell lines and a tumor xenograft model 58. NC treatment did not have liver or kidney toxicity in mouse model 58. This group further identified that NC suppressed Mcl-1 protein level via lysosome-dependent degradation in oral squamous cell carcinoma 59. These reports suggest that NC might be a putative agent against oral cancer 58. NC exposure exhibited growth inhibition of AML via induction of cell cycle arrest and apoptosis 60. NC elevated the expression of p27 and Bax, and reduced the level of Cyclin B1, CDK1 and Bcl-2, as well as inactivated PARP in AML cells 60. Moreover, NC-induced cell growth inhibition in AML cells is partly due to inactivation of the phosphorylation of Akt and ERK 60. Similarly, NC induced erythroid differentiation and apoptosis in CML via regulation of c-Myc-miRNAs axis 61. In addition, NC repressed cell proliferation and enhanced apoptotic death through elevation of p53 in NPC cells 62. Recently, NC was reported to inhibit cell proliferation and invasion through suppression of YAP expression in prostate cancer cells 63. We believe the tumor suppressive activity of NC will be exhibited in a range of human malignancies.

## Conclusions

NC has been characterized as a potent anti-tumor agent in a wide spectrum of human cancers via various molecular mechanisms (Table 1). In this regard, NC exhibits tumor suppressive functions through targeting numerous signaling pathways. It is noted that NC could have nephrotoxicity due to high-affinity of OCT2 and MATE1 to NC in kidney and have its hepatotoxicity 29, 64. However, multiple questions should be answered before NC will be used in clinical trial. For example, NC is absolutely safe for using in human cancer patients? How can NC be delivered to specific organs with tumors? How to improve the NC bio-solubility in human body? What are the detailed and specific molecular mechanisms of NC in different types of human cancers? We believe that answering these questions will promote NC to be used in clinic trials in the near future.

## Figures and Tables

**Table 1:** Summary of the functions of nitidine chloride in human cancers.

Cancer type	Function	Targets	Reference
Breast cancer	Inhibits growth and induces cell cycle arrest; reduces migration and invasion; inhibits metastasis.	Downregulates c-Src/FAK; MMP-9, MMP-2, c-Src, FAK, MAPK, RhoA, Rac1, AP, Bcl-2, smoothened, patched, Gli1, Gli2, Snail, Slug, Zeb1, Nanog, Nestn, Oct-4, and CD44; upregulates p53, p21, Bax, cleaved caspase-9 and -3, and PARP.	3, 9, 18
Liver cancer	Inhibits cell growth and induces apoptosis and cell cycle arrest.	Decreases JAK1/STAT3, cyclinD1, CDK4, Bcl-2, ERK, SHH, TOP1 and TOP2A; upregulates p21, p53, Bax, and caspase-3 and p21.	20, 21, 26
Ovarian cancer	Inhibits proliferation, migration, invasion, and induced apoptosis.	Downregulates Skp2, MMP-2, MMP-9, ERK, pAkt, Bcl-2. Increases the expression of Fas, FADD, caspase-8 and caspase-3.	31, 32, 38-40
Renal cancer	Suppresses invasion and metastasis; Triggers apoptosis.	Inhibits AKT signaling pathway, down-regulates MMP-2 and MMP-9. inhibited phosphorylation of ERK and Akt, upregulates p53, Bax, cleavage caspase-3, and cleavage PARP, and downregulates Bcl-2, caspase-3 and PARP,	43, 44
Glioblastoma	Inhibits cell viability, migration and invasion, and induces cell cycle arrest.	Suppresses pAkt, mTOR, pDok2, GSK3-b; increases cleaved PARP and cleaved caspase 3.	48, 49
Osteosarcoma	Inhibits proliferation, migration, invasion and EMT, induces the apoptosis.	Upregulates cleaved caspase-3, cleaved caspase-9, E-cadherin and Bax. Downregulates pro-caspase-3, pro-caspase-9, Bcl-2; Akt/GSK-3/Snail, SIN1, N-cadherin, vimentin, and fibronectn.	53-55
Colorectal cancer	Inhibits proliferation, enhances apoptosis.	Increases Bax, p53, cleaved caspase-3 and -9. Decreases Bcl-2, and pERK.	56
Gastric cancer	Inhibits angiogenesis and metastasis.	Inhibits STAT3 activation, cyclin D1, Bcl-xL, and VEGF.	6
Oral cancer	Inhibits cell viability, and enhanced apoptosis.	Downregulates STAT3 signaling pathway, suppresses Mcl-1 level.	58, 59
Acute myeloid leukemia	Inhibits cell growth, induces cell cycle arrest and apoptosis;	Increases p27, Bax; Decreases Cyclin B1, CDK1, Bcl-2, pAkt, and ERK.	60
Chronic myeloid leukemia	Induces erythroid differentiation and apoptosis.	Targets c-Myc-miRNAs axis.	61
Nasopharyngeal carcinoma	Inhibits proliferation, and enhances apoptosis.	Upregulates p53.	62
Prostate cancer	Inhibits cell proliferation and invasion.	Suppresses YAP.	63

## Abbreviations

AP: activator protein
AML: acute myeloid leukemia
Bcl-2: B-cell lymphoma 2
CDK4: cyclin-dependent kinase 4
CML: chronic myeloid leukemia
CRC: colorectal cancer
CSC: cancer stem cells
CYPs: cytochrome P450 enzymes
EMT: epithelial mesenchymal transition
ERK: extracellular regulated protein kinases
FADD: Fas-associating protein with a novel death domain
FAK: focal adhesion kinase
FasL: Fas ligand
GBM: Glioblastoma multiforme
GC: gastric cancer
Gli: glioma-associated oncogene homolog
GSK-3β: Glycogen synthase kinase-3β
HCC: hepatocellular carcinoma
HIV: human immunodeficiency viruses
HH: hedgehog
JAK1: Janus kinase 1
LC: Liver cancer
MAPK: mitogen-activated protein kinase
MATE1: multidrug and toxin extrusion 1
MCL1: myeloid cell leukemia 1
MMP: Matrix metalloproteinase
mTOR: mammalian target of rapamycin
NC: Nitidine chloride
NPC: nasopharyngeal carcinoma
OC: Ovarian cancer
OCT1: organic cation transporter 1
PARP: poly ADP-ribose polymerase
PDGF: Platelet derived growth factor
RCC: renal cell carcinoma
SCF: Skp1-Cullin1-F-box complex
SHH: sonic hedgehog
Skp2: S-phase kinase associated protein 2
STAT3: signal transducer and activator of transcription 3
TOP1: topoisomerase 1
VEGF: vascular endothelial growth factor
ZEB1: Zinc finger E-box-binding homeobox 1
AP: activator protein
AML: acute myeloid leukemia
Bcl-2: B-cell lymphoma 2
CDK4: cyclin-dependent kinase 4
CML: chronic myeloid leukemia
CRC: colorectal cancer
CSC: cancer stem cells
CYPs: cytochrome P450 enzymes
EMT: epithelial mesenchymal transition
ERK: extracellular regulated protein kinases
FADD: Fas-associating protein with a novel death domain
FAK: focal adhesion kinase
FasL: Fas ligand
GBM: Glioblastoma multiforme
GC: gastric cancer
Gli: glioma-associated oncogene homolog
GSK-3β: Glycogen synthase kinase-3β
HCC: hepatocellular carcinoma
HIV: human immunodeficiency viruses
HH: hedgehog
JAK1: Janus kinase 1
LC: Liver cancer
MAPK: mitogen-activated protein kinase
MATE1: multidrug and toxin extrusion 1
MCL1: myeloid cell leukemia 1
MMP: Matrix metalloproteinase
mTOR: mammalian target of rapamycin
NC: Nitidine chloride
NPC: nasopharyngeal carcinoma
OC: Ovarian cancer
OCT1: organic cation transporter 1
PARP: poly ADP-ribose polymerase
PDGF: Platelet derived growth factor
RCC: renal cell carcinoma
SCF: Skp1-Cullin1-F-box complex
SHH: sonic hedgehog
Skp2: S-phase kinase associated protein 2
STAT3: signal transducer and activator of transcription 3
TOP1: topoisomerase 1
VEGF: vascular endothelial growth factor
ZEB1: Zinc finger E-box-binding homeobox 1
